# Response Prediction to Neoadjuvant Chemotherapy: Comparison between Pre-Therapeutic Gene Expression Profiles and *In Vitro* Chemosensitivity Assay

**DOI:** 10.1371/journal.pone.0066573

**Published:** 2013-06-24

**Authors:** Christian F. Singer, Florian Klinglmüller, Rembert Stratmann, Christine Staudigl, Anneliese Fink-Retter, Daphne Gschwantler, Samir Helmy, Georg Pfeiler, Anne Catharina Dressler, Christian Sartori, Martin Bilban

**Affiliations:** 1 Department of Gynecology and Obstetrics and Comprehensive Cancer Center, Medical University of Vienna, Vienna, Austria; 2 Institute for Medical Statistics and Informatics, Medical University of Vienna, Vienna, Austria; 3 DCS Innovative Diagnostik-Systeme GmbH, Hamburg, Germany; 4 Department of Laboratory Medicine, Medical University of Vienna, Vienna, Austria; King Faisal Specialist Hospital & Research center, Saudi Arabia

## Abstract

Although the use of (neo-)adjuvant chemotherapy in breast cancer patients has resulted in improved outcome, not all patients benefit equally. We have evaluated the utility of an *in vitro* chemosensitivity assay in predicting response to neoadjuvant chemotherapy. Pre-therapeutic biopsies were obtained from 30 breast cancer patients assigned to neoadjuvant epirubicin 75 mg/m2 and docetaxel 75 mg/m2 (Epi/Doc) in a prospectively randomized clinical trial. Biopsies were subjected to a standardized ATP-based Epi/Doc chemosensitivity assay, and to gene expression profiling. Patients then received 3 cycles of chemotherapy, and response was evaluated by changes in tumor diameter and Ki67 expression. The efficacy of Epi/Doc *in vitro* was correlated with differential changes in tumor cell proliferation in response to Epi/Doc *in vivo* (p = 0.0011; r = 0.73670, Spearmańs rho), but did not predict for changes in tumor size. While a pre-therapeutic gene expression signature identified tumors with a clinical response to Epi/Doc, no such signature could be found for tumors that responded to Epi/Doc *in vitro*, or tumors in which Epi/Doc exerted an antiproliferative effect *in vivo*. This is the first prospective clinical trial to demonstrate the utility of a standardized *in vitro* chemosensitivity assay in predicting the individual biological response to chemotherapy in breast cancer.

## Introduction

Neo-adjuvant chemotherapy has traditionally been used to treat locally advanced and initially inoperable breast cancer, but is now increasingly also used in the treatment of smaller tumors [1]. One of the main reasons for applying a systemic therapy prior to rather than after curative surgery is the potential reduction in the size of a malignant tumor which is thought to permit less invasive curative surgery. In addition, women in whom a clinical, and more so, a pathological remission can be achieved prior to surgery, enjoy an improved outcome [2]. Unfortumately, while in many cases a clinically meaningful remission can be achieved, not all patients benefit equally. Some tumors even increase in size despite ongoing chemotherapy, thereby suggesting *a priori* resistance [3]. Nevertheless, neoadjuvant and adjuvant chemotherapy is still applied empirically, since until today, no clinical tests exists that would allow to reliably predict response to and benefit from a particular chemotherapy.

The difficulty to adequatly measure tumor response has hampered resarch in the field of neoadjuvant systemic therapy considerably. Clinical evaluation of tumor response to neoadjuvant treatment is usually performed by caliper, ultrasound, or mammography [4]. While these techniques are readily available and can easily be applied, they are subject to considerable methodological inaccuracy, and are not sensitive enough to detect early biological effects such as proliferative changes and tumor cell apoptosis – both cellular responses that are thought to be underlie tumor shrinkage [5], [6].

Ki-67 is a nuclear antigen which is expressed during G_1_, S and G_2_ phase and which has been established as a proliferation marker in many solid tumors, since it can be detected immunohistochemically in a semiquantitative manner. A high level of pre-therapeutic proliferation activity in breast cancer has been associated with poor prognosis, but is also predictive for response to preoperative chemotherapy [7]. Furthermore, changes in the relative proportion of Ki-67 positive cells can be observed during preoperative chemotherapy and correlate well with clinical and pathological response in early breast cancer [8], [9].

Several studies have found pre- to post-therapy changes in Ki67 to be strong and independent predictors of disease-free and relapse-free survival, and both pre- and post therapy values have been demonstrated to be significant independent predictors for overall survival on multivariate analysis [10]–[13]. It is mainly because of these reasons that tumor response in many neo-adjuvant endocrine and chemotherapy trials is now mainly evaluated by Ki67 immunohistochemistry.

While the individual response of a specific chemotherapy in a particular patient can be assessed *in vivo* by measuring biological parameters or by following clinical endpoints, we are still far from being able to predict an individual response to a given regimen, or to single out the one particular antineoplastic drug that will be the most effective out of a number of other substances. This dilemma has lead to the utilization of gene expression arrays, in which specific gene expression signatures have been proposed to predict response to selective chemotherapies [14], [15].

Another strategy is the development of *in vitro* drug assays, in which short-term cultures of breast cancer-derived cell suspensions are subjected to a number of cytotoxic drugs *in vitro*. Among these, the microplate adenosine triphosphate (ATP)-based tumor chemosensitivity assay (ATP-TCA) has gained particular interest for *ex vivo* chemosensitivity testing of native nonhematological tumors [16]. It has been hypothesized that the most effective drug *in vitro* would also result in a superior response *in vivo.* Indeed, in primary ovarian cancers, the ATP-TCA has been reported to accurately predict both clinical response and survival: In two prospectively designed clinical trials in patients with heavily pretreated ovarian cancer, chemotherapy regimen that were individually selected by the ATP-TCA have been found to triple the response rates and nearly double the survival compared to empirically chosen regimens [17], [18]. However, this has never been confirmed in breast cancer.

We have used the ATP-TCA in order to measure the effect of Epi/Doc on the survival of tumor cell suspensions *in vitro*, and compared the results with the *in vivo* effect of the same regimen on Ki67 reduction.

## Materials and Methods

### Patients and Treatment Intervention

This prospective clinical investigation followed Good Clinical Practice guidelines, and all patients provided written informed consent prior to enrolment. The study was approved by the IRB of the Medical University of Vienna and was conducted in Austria. The clinical investigation has been conducted according to the principles expressed in the Declaration of Helsinki. Eligible patients had to have histologically proven invasive breast cancer stage I-III, and the presence of distant metastasis as evaluated by chest x-ray, liver sonography and bone scan had to be ruled out prior to study entry. All patients received three cycles of epirubicin 75 mg/m^2^ IV followed by docetaxel 75 mg/m^2^ (1-hour IV infusion) on day 1 and granulocyte colony-stimulating factor (G-CSF) 5 µg/kg/d subcutaneously on days 3 to 10 (filgrastim, Neupogen; Amgen, Thousand Oaks, CA).

**Figure 1:**
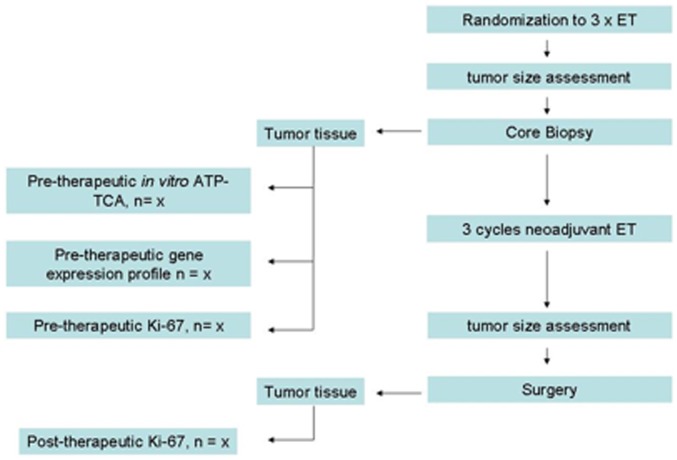
Study flow chart.

Tumor diameter were measured by ultrasound before systemic therapy and immediately prior to curative surgery, and clinical responses were evaluated according to RECIST criteria [19]. A reduction in the longest tumor diameter of >30% was required for classifying a tumor as responsive. Pre-treatment tumor samples were obtained by three to six 14-gauge core needle biopsies, and were equally split in three portions: one portion was used for histological confirmation of invasive breast cancer and for evaluation of Ki67 protein expression by IHC. The second portion was subjected to ATP-TCA, while the third portion was immediately snap-frozen in dry ice for gene expression profiling. Post-treatment tumor samples for Ki67 determination were obtained during curative surgery, which was performed within 2 to 4 weeks after the last scheduled chemotherapy cycle. Tumors which had a decrease in Ki67 expression of >30% in response to Epi/Doc were considered sensitive. The procedural flow is shown in figure 1.

### ATP-TCA Chemosensitivity Testing

Primary human breast cancer cells were isolated from pre-treatment tumor samples, and ATP-TCA was performed as previously described [20]. In brief, 10 000–20 000 tumor cells per well were seeded in tumor cell-supporting growth medium in 96-well microtiter plates. Epirubicin and Docetaxel were used in combination at test drug concentrations (TDCs) of 200, 100, 50, 25, 12.5 and 6.25% (with 100% TDC corresponding to peak plasma concentrations). All tests were performed in triplicates. Tumor cells were lysed after 7 days of incubation and the amount of cellular ATP was determined with a luciferin/luciferase reaction. Cell preparation and ATP-TCA were performed using commercially available reagents (TCA-100, DCS, Hamburg, Germany). Luminescence was measured with either a LB953 luminometer (Berthold Technologies, Bad Wildbad, Germany) or a MPL2 Microplate Luminometer (Berthold Detection Systems, Pforzheim, Germany). Chemosensitivity across the tested concentration range was evaluated by using the Sensitivity Index (“SI”) [21]. The SI is obtained by measuring the area under the inhibition curve for multiple dilutions of the effective chemotherapy dose. A low SI indicates good chemosensitivity to a particular antineoplastic compound *in vitro*, while a high SI value describes poor chemosensitivity.

### RNA Extraction, Amplification and GeneChip Hybridization

RNA extraction, amplification and GeneChip hybridization was performed according to the manufactureŕs protocol (Affymetrix Inc., Santa Clara, CA). In brief, double-stranded cDNA was synthesized from one patient by a chimeric oligonucleotide with an oligo-dT and a T7 RNA polymerase promoter at a concentration of 100 pmol/µl. Reverse transcription was performed as recommended by Affymetrix by using commercially available reagents (Invitrogen). The reaction products were cleaned by phenol-chloroform extraction before biotin-labelling and an approximately 250-fold amplification were performed. 10 µg of each of the 6 labelled cRNA samples was then hybridized onto a separate Affymetrix U133 GeneChip® Set. The set consists of two GeneChip® arrays and contains almost 45,000 probe sets representing more than 39,000 transcripts derived from approximately 33,000 well-substantiated human genes. The set design uses sequences selected from GenBank®, dbEST, and RefSeq. Prehybridization, hybridization, washing and staining with streptavidin-phycoerythrin were carried out according to the manufactureŕs protocol. Antibody amplification was accomplished using a biotin-linked antistreptavidin antibody (Vector Laboratories, Buringame, CA). The goat immunoglobulin G-blocking (Sigma, St Louis, MO) antibody was used for blocking unspecific binding. The arrays were then stained and washed as recommendet by Affymetrix, before being scanned on an Affymetrix GeneChip Scanner (Agilent, Palo Alto, CA). The gene expression data presented in our manuscript have been deposited in a publicly available data base (Gene Expression Omnibus) and can be accessed under the following weblink: http://www.ncbi.nlm.nih.gov/geo/query/acc.cgi?acc=GSE29561.

### Statistical Analysis

Microarray data were preprocessed using quantile normalization and RMA [22]. In order to test the null hyothesis of no differential expression between tumors that showed considerable size reduction after treatment and those that did not, for each gene Welch‘s two sample t-test was computed. Marginal p-values were adjusted using methods previously described in order to provide control over the local false discovery rate (locFDR). A cut-off of 0.15 was considered to achieve similar control as conventional.05 α-levels when using the false discovery rate. For the sake of exploration, genes with a locFDR of up to.25 were considered. All computations were implemented using R (http://cran.r-project.org) and Bioconductor (http://www.bioconductor.org) [23], [24].

## Results

### Patient Characteristics

Of the 30 patients who were randomized, 26 received three cycles of Epi/Doc according to protocol. Their clinico-pathological characteristics are shown in table 1. Three patients (12%) experienced a pCR (as defined by the absence of invasive tumor components), while invasive tumor components were detected in the surgical specimen in the remaining 23 cases. In all of these cases, both pre- and post-therapeutic tumor measurements and Ki67 values were available. The ATP-TCA could be peformed in 18 cases. However, due to the fact that some patients experienced a pCR post-therapeutic Ki67 was not available in all cases. Results for both, differential pre- vs post-treatment Ki67 values, and ATP-TCA, were evaluated in 16 cases. Pre-treatment gene expression profiles were available in 22 of the 26 patients who had received Epi/Doc according to protocol.

**Table 1:** Patient characteristics.

Characteristics		n = 26 (100%)
Median age in years (range)		47 (33–77)
Menopausal stage		
	Premenopausal	16 (62%)
	Postmenopausal	10 (38%)
Tumor stage, n (%)		
	T1	10 (38%)
	T2	9 (35%)
	T3	5 (19%)
	T4	2 (8%)
Nodal stage, n (%)		
	Negative	13 (50%)
	Positive	12 (46%)
	Not evaluated	1 (4%)
Estrogen receptor status		
	Positive	16 (62%)
	Negative	10 (38%)
Progesteron receptor status		
	Positive	13 (50%)
	Negative	13 (50%)
HER-2 overexpression		
	Positive	6 (23%)
	Negative	20 (77%)
Grading		
	G1-2	10 (38%)
	G3	16 (62%)

### In vitro Chemosensitivity of Epi/Doc in the ATP-TCA

**Figure 2:**
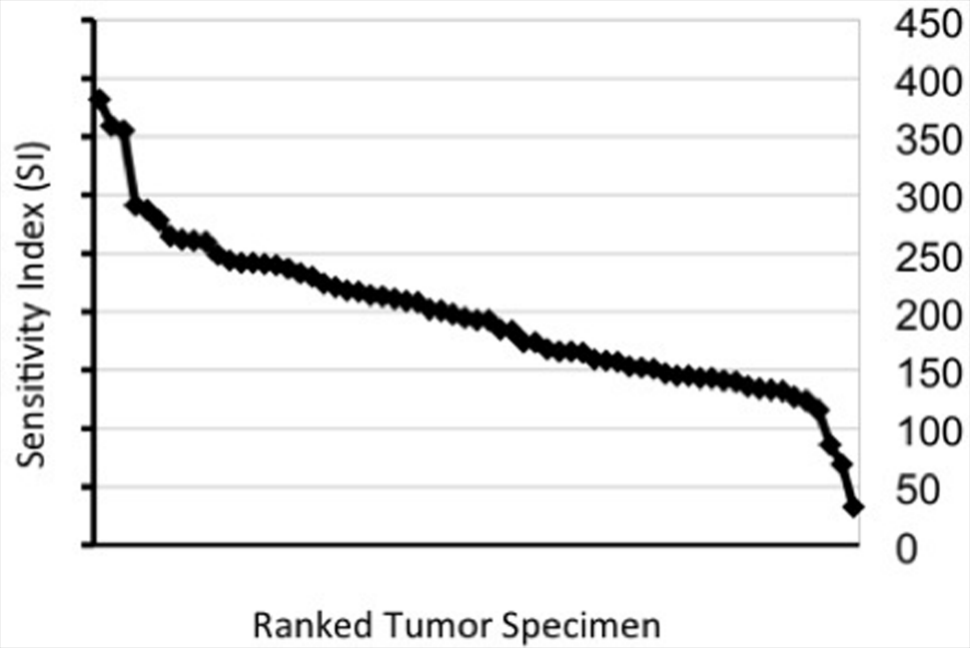
Sensitivity index (SI) describing i*n vitro* chemosensitivity to Epi/Doc over a concentration range of a panel of 65 breast cancer samples. Tumor samples are arranged from left to right according to increasing SI.

18 freshly isolated pre-therapeutic tumor samples from patients subsequently undergoing neoadjuvant Epi/Doc were subjected to Epi/Doc in the ATP-TCA as described in Materials and Methods. Chemosensitivity along a dilution series of the cytotoxic drug combination was evaluated for each sample by using the sensitivity index (SI), as previously described by Kurbacher *et al.* ATP-TCA data from 47 additional tumor cell isolates from breast cancer patients not enroled into the trial were also available and were included for figure 2. The sensitivity curve depicts individual SI values ordered by increasing in vitro sensitivity to Epi/Doc and demonstrates a highly heterogenous sensitivity to Epi/Doc.

### Effect of 3 Cycles of Neoadjuvant Epi/Doc on Tumor Size and Intratumoral Ki67 Expression

Three cycles of Epi/Doc lead to a reduction in tumor size of >30% in the longest tumor diameter in 11 out of 23 patients (48%). In the 23 tumors in which intratumoral Ki67 protein expression was measured before and after neoadjuvant treatment with 3 cycles of Epi/Doc, we found a reduction in Ki67 of >30% in 10 cases (43%). Interestingly, although both measurements yielded comparable rates in terms of antitumoral effects (response vs non-response) in vivo, we did not find a significant association when the relative reductions in tumor size (mm) were correlated with the relative reduction in Ki67 expression (%) (r = 0.1797, p = 0.3176; Kendall tau, data not shown).

### Effect of Epi/Doc on Tumor Cell Proliferation in vitro (ATP-TCA)

**Figure 3:**
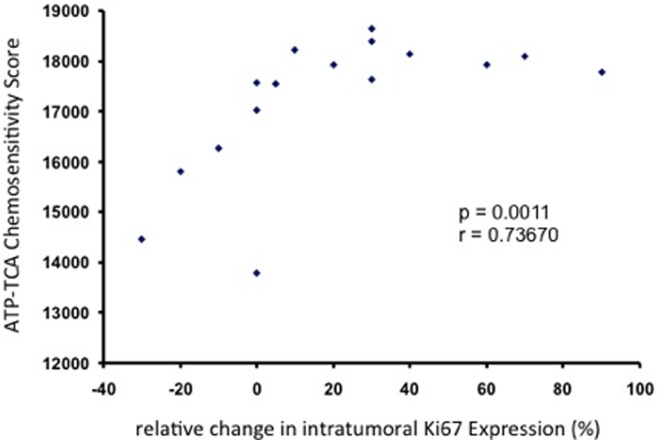
Correlation between the relative changes (%) in intratumoral Ki67 expression in response to 3 cycles of neoadjuvant Epi/Doc, and in vitro chemosensitivity to Epi/Doc as measured by ATP-TCA.

We then investigated the response to Epi/Doc in short term primary cultures which had been obtained from malignant breast tumors of individual patients who had subsequently undergone neoadjuvant Epi/Doc treatment. ATP-TCA results were available in 16 cases, and 9 (56%) tumor tisues were found to be responsive according to the pre-defined cut-off values described in Materials and Methods. Tumor cells which responded to Epi/Doc in the ATP-TCA *in vitro* also responded to 3 cycles of Epi/Doc *in vivo* (p = 0.041, Fisheŕs Exact Test, table 2). In addition, the antiproliferative effect of Epi/Doc in the ATP-TCA assay was significantly correlated to the decrease in Ki67 protein expression in the same patients (p = 0.001, r = 0.737, Kendalĺs Tau, figure 3). When we investigated whether tumor sensitivity to Epi/Doc determined by ATP-TCA was able to predict a clinically meaninful remission in response to neoadjuvant Epi/Doc if tumor response was measured by breast ultrasound, we did not observe a comparable pattern (p = 0.999, Fisheŕs Exact Test, table 3).

**Table 2:** 2×2 table showing tumor response to 3 cycles of neoadjuvant Epi/Doc as defined in Materials and Methods and chemosensitivity to the dichotomized Ki67 change (<30% vs >30%).

Ki67 change *in vivo*	ATP-TCA		
	no	yes	sum
No	6	2	8
Yes	1	7	8
Sum	7	9	16

r = 0.041, Fisheŕs Exact Test.

**Table 3:** 2×2 table showing tumor response to 3 cycles of neoadjuvant Epi/Doc as defined in Materials and Methods and chemosensitivity to Epi/Doc *in vitro* (ATP-TCA).

Change in tumor size	ATP-TCA		
	no	yes	sum
no	3	5	8
yes	5	5	10
Sum	8	10	18

r = 0.664, Fisheŕs Exact Test.

### Gene Expression Profile and Response to Epi/Doc in vitro and in vivo

**Figure 4:**
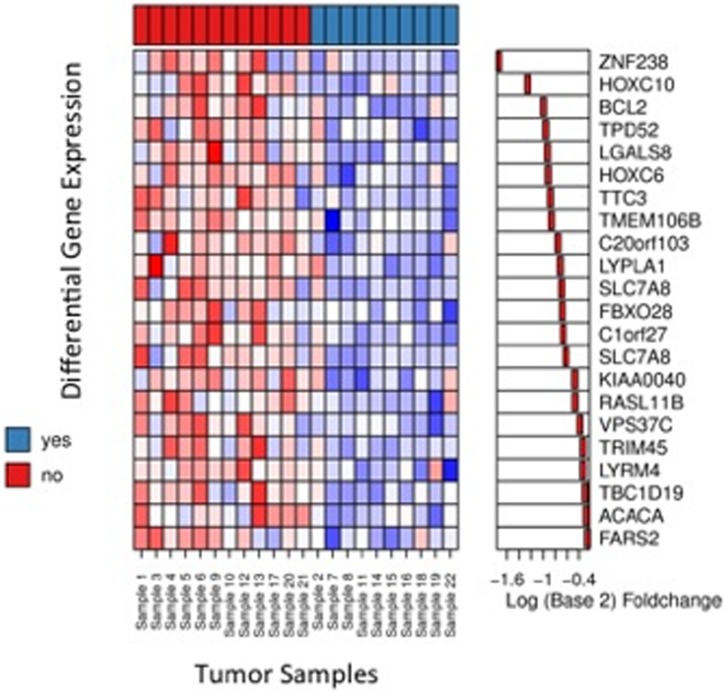
Heatmap of Genes with local FDR less than 0.25. Red corresponds to high, blue to low signals. Genes are annotated using gene symbols. Color code at the top indicates treatment success. Corresponding fold changes are depicted in the right slide panel.

We then investigated whether we could identify gene expression profiles in the initial core biopsy that would permit to a) predict a clinically meaningful response to Epi/Doc in terms of tumor size reduction of >30%, b) predict a profound reduction in intratumoral Ki67 protein expression, and c) predict an *in vitro* response to Epi/Doc in the ATP-TCA. When the analyses were carried out as described in Materials and Methods, we were unable to identify a gene expression signature that was associated with a reduction in Ki67 *in vivo*, or chemosensitivity *in vitro* in our training set. We did, however, identify a set of 5 genes that were significantly up-regulated (locFDR<15%) in chemosensitive tumors. Table 4 provides annotated results for genes which show expression differences with a locFDR level of 25% and less. A Heatmap of the corresponding expression profiles is shown in figure 4.

**Table 4:** Genes expression of genes with a local false discovery rate (FDR) <0.25 for the discrimination between tumors with a clinically meaningful reduction in tumor size under neoadjuvant Epi/Doc from those which do not benefit from therapy.

Gene.Name	Gene.Sbol	locFDR	logFC
zinc finger protein 238	ZNF238	0.18	−1.74
homeobox C10	HOXC10	0.18	−1.26
B-cell CLL/lymphoma 2	BCL2	0.24	−0.99
tumor protein D52	TPD52	0.25	−0.96
lectin, galactoside-binding, soluble, 8	LGALS8	0.19	−0.93
homeobox C6	HOXC6	0.21	−0.92
tetratricopeptide repeat domain 3	TTC3	0.15	−0.88
transmembrane protein 106B	TMEM106B	0.22	−0.86
chromosome 20 open reading frame 103	C20orf103	0.12	−0.76
lysophospholipase I	LYPLA1	0.20	−0.72
solute carrier family 7 (cationic amino acid transporter, y+ system), member 8	SLC7A8	0.14	−0.69
F-box protein 28	FBXO28	0.24	−0.68
chromosome 1 open reading frame 27	C1orf27	0.12	−0.66
solute carrier family 7 (cationic amino acid transporter, y+ system), member 8	SLC7A8	0.19	−0.62
KIAA0040	KIAA0040	0.24	−0.47
RAS-like, family 11, member B	RASL11B	0.19	−0.46
vacuolar protein sorting 37 homolog C (S. cerevisiae)	VPS37C	0.15	−0.38
tripartite motif-containing 45	TRIM45	0.23	−0.33
LYR motif containing 4	LYRM4	0.24	−0.33
TBC1 domain family, member 19	TBC1D19	0.18	−0.30
acetyl-Coenzyme A carboxylase alpha	ACACA	0.17	−0.28
phenylalanyl-tRNA synthetase 2, mitochondrial	FARS2	0.21	−0.26

## Discussion

Pathological complete response (pCR) following neoadjuvant chemotherapy is seen in 3–26% of patients, and is a good predictor of survival [25]. Although the majority of neoadjuvant regimen do not achieve pCR, tumors can still respond in a clinically meaningful way. Response to neoadjuvant chemotherapy has traditionally been evaluated by ultrasound, mammography, or caliper measurements. The value of these techniques in predicting response, however, is now being increasingly challenged because they do not provide adequate information about the biological activity of a particular tumor [4]. It is not uncommon to experienced surgeons to discover the absence of viable invasive tumor cells in the specimen of a breast cancer that had not appreciably changed its size under systemic therapy. Other diagnostic techniques such as FDG-PET, which measures the uptake of labeled glucose into a tumor, are thus better suited to evaluate biological activity at a given point in time and are currently evaluated [26], [27]. An alternative to evaluate the presence of viable tumor cells is by measuring the readily available immunohistochemical analysis of the nuclear antigen Ki67 [28]. There is now increasing interest in the nuclear proliferation antigen Ki67, since allows to distinguish the relatively chemo-resistant luminal A from the more sensitive luminal B breast cancer subtype [29]. It is now well established that higher levels of post-treatment Ki67 values are associated with poorer survival in breast cancer, while higher pre-therapeutic intratumoral Ki67 levels are associated with improved response [30], [31].

The value of Ki67 in assessing neoadjuvant response has recently been demonstrated by Dowsett *et al.*, who reported that a 2 week pre-operative treatment with the aromatase inhibitor anastrozole was more effective in reducing intratumoral Ki67 levels than tamoxifen, or a combination of anastrozole and tamoxifen, in postmenopausal receptor-positive breast cancer patients (“IMPACT” trial). Their findings exactly matched the results of the much larger adjuvant ATAC trial, in which disease-free survival in response to the same three regimen was chosen as primary endpoint. Interestingly, the group also used ultrasound to measure tumor size alterations in response to the three treatment strategies, and – similar to our results – also failed to detect differential changes across the three arms, thus suggesting that ultrasound might simply not be sensitive enough to measure biological response [32]. This hypothesis is, however, somewhat challenged by the fact that, similar to Chang *et al*., we also identified a particular gene expression signature that is characteristic of chemosensitive tumors when measured by ultrasound, while no such pattern was seen when the tumor response was evaluated by Ki67 expression [15].

Tumor shrinkage is a robust, albeit relatively insensitive parameter which was readily available also in tumors with pCR. In these tumors, however the relatively sensitive Ki67 values were no longer available and a differential Ki67 expression could thus not be determined. This circumstance reduced the number of evaluable tumors and might thus have impacted on the statistical significance. It is well possible that with a larger sample size, a particular expression profile might have emerged. Alternatively, individual pre- and posttreatment Ki67 measurements can be difficult to assess, and the measurement of a differential Ki67 expression might result in even greater imprecision, which could also have contributed to our inability to identify a Ki67 response signature.

We were also unable to identify a ATP-TCA chemosensitivity-associated expression profile by using the human genome Affymetrix U133 GeneChip® set which comprises more than 33.000 well characterized genes. This is in sharp contrast to two recent publications in non small-cell lung cancer (NSCLC) and in platinum-pretreated ovarian cancer, in which the RT-PCR based expression signature derived from a panel of only 23 resistance-associated genes was associated with ATP-TCA-measured chemosensitivity [33], [34]. In our case, the genetic heterogeneity of malignant breast tumors, the fact that the tissue preparation that precedes the ATP-TCA alters the cellular composition of the initial tumor, and the relatively small sample size may have all contributed to the absence of a clear chemosensitivity signature.

Our work has several limitations: The difficulty in obtaining sufficient pre-therapeutic tissue by core biopsy has allowed us to perform both ATP-TCA and gene expression profiling in only 16 out of 30 patients, thus resulting in a comparatively small number of samples which makes this a pilot trial. Also, while tumor response was evaluated according to RECIST criteria, the 30% cut-off level of for response to Ki67 was chosen arbitrarily to allow for equal group sizes. In addition, only one chemotherapy regimen (Epi/Doc) was evaluated *in vivo*. While we have also generated *in vitro* data for Epirubicin, Docetaxel, and for several other chemotoxic drugs individually (data not shown), we do not know whether their efficacy in the assay also translates into a comparable reduction in Ki67 *in vivo.* Furthermore, while it is tempting to speculate that the most effective drug in the ATP-TCA is also the most potent drug *in vivo*, this requires further prospective evaluation.

Finally, in contrast to analytical tools such as gene expression profiling or proteomic analysis, the ATP-TCA offers little mechanistic insight into why a particular drug is effective in one tumor but fails to do so in another for it reflects the net sum of cellular responses to chemotherapy. Nevertheless, the results of our pilot trial suggest, for the first time, the utility of a standardized *in vitro* chemosensitivity assay in predicting the individual biological response to chemotherapy in breast cancer patients in a prospective setting.
